# Evaluating acupuncture and standard care for pregnant women with back pain: the EASE Back pilot randomised controlled trial (ISRCTN49955124)

**DOI:** 10.1186/s40814-016-0107-6

**Published:** 2016-12-12

**Authors:** Annette Bishop, Reuben Ogollah, Bernadette Bartlam, Panos Barlas, Melanie A. Holden, Khaled M. Ismail, Sue Jowett, Martyn Lewis, Alison Lloyd, Christine Kettle, Jesse Kigozi, Nadine E. Foster, the EASE Back study team, Jo Bailey, Ruth Beardmore, Helen Duffy, Michelle Robinson, Julie Young

**Affiliations:** 1 Research Institute for Primary Care & Health Sciences, Keele University, Keele, Staffordshire ST5 5BG UK; 2 School of Health and Rehabilitation, Keele University, Keele, UK; 3 Birmingham Centre for Women’s and Children’s Health, College of Medical and Dental Sciences, University of Birmingham, Birmingham, UK; 4 School of Health and Population Sciences, University of Birmingham, Birmingham, UK; 5 Manchester Metropolitan University, Manchester, UK; 6 Faculty of Health Sciences, Staffordshire University, Stafford, UK; 7 Institute of Metabolism and Systems Research, University of Birmingham, Birmingham, UK; 8 Health Economics Unit, University of Birmingham, Birmingham, UK

**Keywords:** Acupuncture, Pregnancy, Low back pain, Pelvic girdle pain, Physiotherapy, RCT, Pilot

## Abstract

**Background:**

Low back pain (LBP) and pelvic girdle pain (PGP) during pregnancy are common and often accepted as a ‘normal’ part of pregnancy. Many women receive little in the way of treatment, and yet pain interferes with sleep, daily activities and work and leads to increasing requests for induction of labour or elective caesarean section. The aim of this study was to assess the feasibility of a full RCT evaluating the benefit of acupuncture for pregnancy-related back pain.

**Methods:**

This study is a single-centre, three-arm pilot RCT in one large maternity unit and associated antenatal and physiotherapy clinics. Women were eligible if they had pregnancy-related LBP with or without PGP. Exclusions included a history of miscarriage, high risk of early labour or pre-eclampsia, PGP only and previous acupuncture. Interventions were standard care (SC): a self-management booklet with physiotherapy if needed. SC+TA: the booklet and physiotherapy comprising true (penetrating) acupuncture, advice and exercise. SC+NPA: the booklet and physiotherapy comprising non-penetrating acupuncture, advice and exercise. Remote telephone randomisation used a 1:1:1 allocation ratio stratified by gestational weeks. Three measures of pain/function were compared to inform the primary outcome measure in a full RCT: the Pelvic Girdle Questionnaire (PGQ), Oswestry Disability Index (ODI) and 11-point 0–10 numerical rating scale for pain. Analysis focused on process evaluation of recruitment, retention, descriptive information on outcomes, adherence to treatment, occurrence of adverse events and impact of physiotherapist training.

**Results:**

One hundred twenty-five women were randomised (45% of those eligible) between April and October 2013; 73% (*n* = 91) provided 8-week follow-up data. Three of six recruitment methods accounted for 82% of total uptake: screening questionnaire at the 20-week scan, community midwives issuing study cards, and self-referral following local awareness initiatives. Physiotherapists’ self-confidence on managing pregnancy-related LBP improved post training. The PGQ is suitable as the primary outcome in a full trial. The average number of treatment sessions in both SC+TA and SC+NPA was six (in line with treatment protocols). No serious adverse events attributable to the trial treatments were reported.

**Conclusions:**

A full RCT is feasible and would provide evidence about the effectiveness of acupuncture and inform treatment choices for women with pregnancy-related LBP.

**Trial registration:**

ISRCTN49955124

## Background

### Pregnancy-related back pain

Low back pain (LBP) and pelvic girdle pain (PGP) during pregnancy are common. Whilst they can occur separately, many women experience both. Prevalence estimates suggest between 45 and 75% of women experience LBP at some stage during their pregnancy [1, 2] and up to 70% of women experience PGP by late pregnancy [3–5]. LBP, with or without PGP, is referred to as pregnancy-related LBP throughout this paper. Studies have shown that women with pregnancy-related LBP have lower quality of life compared with non-pregnant healthy women [6], and reports suggest that 20 to 23% of women take sick leave because of their pain [2]. Pregnancy-related LBP increases with advancing pregnancy, is usually worse at night and interferes with sleep, daily activities and work [4]. Indeed, an increasing number of affected women request induction of labour or elective caesarean section before the recommended 39th week of gestation [7].

### Current clinical management

Pregnancy-related LBP is commonly accepted as a ‘normal’ part of pregnancy [2, 7], and women are encouraged to believe that their pain is temporary and self-limiting which may not always be the case. Women with pregnancy-related LBP most often report problems to their midwife, but few receive active treatment for the condition. In an Australian study, 71% reported their pain to their maternity carer, but only 25% received any treatment [2].

Most women with pain are advised about self-management strategies [4]; however, some access a range of treatments, including physiotherapy-led exercise, manual therapy, transcutaneous electrical nerve stimulation (TENS), safe pharmacological options (mostly paracetamol), and much less commonly, epidural injections [2, 4, 7]. In the UK National Health Service (NHS), management varies across services and geographical regions. Most women are not referred from their midwives or GPs to other health professionals, such as physiotherapists, for treatment. Where they are offered physiotherapy, some are offered one-off educational classes focusing on self-management advice and others are offered courses of individualised treatment [8].

### Acupuncture

Acupuncture is commonly used for musculoskeletal problems and is recommended within UK national guidelines for the management of persistent non-specific LBP [9]. A small number of studies have evaluated acupuncture for pregnancy-related LBP, which have been summarised in two systematic reviews [4, 10] concluding promising but limited evidence for its use and highlight the need for further high quality trials.

This paper reports a pilot randomised controlled trial (RCT) to test the feasibility of a future, full trial to evaluate the effectiveness of adding acupuncture to standard care (SC) for pregnancy-related LBP. In order to inform the interventions and methods of the pilot RCT, we previously investigated (i) current UK physiotherapy management of pregnancy-related LBP [8], (ii) the views of physiotherapists about using acupuncture for pregnancy-related LBP [11] and (iii) the views of women with pregnancy-related LBP and midwives about a RCT, including recruitment and consent procedures and safety concerns [12].

The specific objectives of the EASE Back pilot trial were to:

- Test the trial procedures, training programme for health care professionals, interventions and outcome measures in women with pregnancy-related LBP.
- Provide data on likely recruitment and follow-up rates for a main trial plus completion rates on key outcomes and an estimate of the standard deviation for the outcome in order to inform the sample size requirements of a full trial.

Specific success criteria for progression to a full trial were not specified a priori, but the findings were discussed with both the Trial Steering Committee and at a multi-disciplinary consensus meeting which included key local and international experts, health professionals and patients who agreed a full trial was feasible.

## Methods

The EASE Back pilot trial is reported in line with the CONSORT [13] and STRICTA guidance [14].

### Trial design and setting

This pilot trial was a single-centre, three-arm parallel pilot RCT based in one large maternity unit and associated antenatal and physiotherapy clinics in Staffordshire, England. Recruitment lasted 27 weeks from April to October 2013, and data were collected at baseline (pre-randomisation) and at 8 weeks post randomisation. Ethical approval was granted by NRES Committee West Midlands—Staffordshire (reference 13/WM/00). The EASE Back pilot trial is registered on the current controlled trials database ISRCTN49955124.

### Participants

Women were eligible for participation in the pilot trial if they met the following criteria: had pregnancy-related LBP defined as self-reported pain in the lumbar area (between the 12th rib and the gluteal fold) with or without PGP; under the care of participating NHS sites; aged 18 years and over; between 13 to 31 weeks gestation (to increase the likelihood that women had time to receive the intervention if they were randomised to SC plus acupuncture and still receive their 8-week follow-up questionnaire before birth); naïve to acupuncture treatment and able to read and communicate in English (to provide full informed consent and to complete the baseline and outcome assessments).

Exclusion criteria were women who had ever received any form of acupuncture previously for any health problem; were at high risk of miscarriage (previously had recurrent miscarriage defined as three or more, abnormalities in the cervix deemed to increase risk of miscarriage, antiphospholipid syndrome, lupus anticoagulant); were at high risk of pre-term labour (previous history of giving birth before 37 weeks gestation, multiple pregnancies, polyhydramnios, pre-term ruptured membranes, history of surgery to the uterine cervix); had diagnosed pre-eclampsia; had a previous history of surgery to the spine or the pelvis; presence of a contra-indication to any of the treatment options (coagulation problems, haemophilia or bleeding disorders, were at increased risk of infection such as skin infections or loss of skin integrity from burns or ulcerations at the site of needling or those with a high needle phobia); had pain in the anterior pelvic girdle region only; and diagnosed with a current urinary tract infection.

These criteria ensured that participants had pregnancy-related LBP with no significant risk factors or contra-indications to acupuncture and hence comparable to those likely to receive these interventions should a full trial demonstrate clinical and cost-effectiveness.

### Identification and recruitment

Six methods of identifying and recruiting women to the pilot trial were tested in order to establish the most successful strategies for a future full trial:

- Method 1: women were identified in routine antenatal clinics by research midwives who determined eligibility, gained consent, conducted baseline assessments and randomisation.
- Method 2: screening questionnaire handed to all women at the 20-week routine ultrasound scan appointment.
- Method 3: research midwives screened referrals to the hospital women’s health physiotherapy service.
- Method 4: research midwives identified potentially eligible women in antenatal clinics, invited them to consider taking part and gave them an EASE Back study card with contact details for the study administrator.
- Method 5: community midwives, obstetricians and GPs identified potentially eligible women, invited them to consider taking part and gave them an EASE Back study card with contact details for the study administrator.
- Method 6: self-referral where women with pregnancy-related LBP who became aware of the pilot trial through a local awareness-raising campaign could directly telephone the EASE Back study administrator. Awareness raising was achieved through study flyers included in standard maternity booking packs and in the 20-week ultrasound scan appointment information, a study-specific website, study flyers and posters displayed in local GP practices, children’s centres and the hospital maternity and women’s health departments. In addition the pilot trial was promoted through local radio including one-off, free, radio interviews about the trial and 3 days of continuous and professionally produced radio infomercials on a local commercial radio station. A local newspaper also ran a free two-page spread and paid advertisements about the study were placed on local buses.

With the exception of method 1, when women contacted the study administrator, a brief initial eligibility check was undertaken over the telephone. Women who were initially eligible were posted study information (letter of invitation, patient information leaflet) and subsequently contacted by a research midwife or research nurse to complete more detailed eligibility checking. Eligible women who were interested in taking part proceeded to a face-to-face appointment with a research midwife or research nurse for confirmation of eligibility, informed consent, baseline assessment and randomisation.

### Randomisation

Eligible women who gave written informed consent to participate were randomised in a 1:1:1 ratio to one of the three treatment arms using randomly varying blocks (sizes 3 and 6) and stratified by gestational weeks (dichotomised to less than or at least 24 weeks). Participants were randomised using remote telephone randomisation provided by a registered Clinical Trials Unit (CTU registration number 36).

A research midwife or research nurse telephoned the CTU administrator who conducted the concealed randomisation process and directly informed the participant about their treatment allocation, in writing. To maintain blinding of participants to the type of acupuncture (true or non-penetrating), treatment allocation letters described the interventions as ‘usual care’ or ‘usual care plus acupuncture’.

### Sample size

In line with pilot trial recommendations [15], EASE Back did not specify a primary outcome nor was a power calculation conducted. The sample size was based on the number of births per year at the participating maternity centre and estimates of the proportion of women likely to be eligible and consent to participate. The maternity service oversaw approximately 6100 births per year, and we estimated that between 50–66% would self-report back pain at some point in their pregnancy and at least 30% would self-report back pain at 13–31 weeks gestation. Thus, we expected that in the region of 600 women would be eligible over a recruitment period of 4 to 5 months with an estimated 25% recruitment rate. To allow for some withdrawal and loss to follow-up, we needed to identify 720 women in order to recruit approximately 180 women and provide follow-up data on 150 women. As an external pilot trial interim analyses and stopping rules were not required.

### Physiotherapists delivering interventions

Fourteen physiotherapists (physical therapists) delivered the interventions. All the physiotherapists were employed in the UK National Health Service (NHS) and had previously completed postgraduate training in the use of acupuncture for musculoskeletal pain conditions, in training programmes that met national and international standards. Prior to the trial, 11 used acupuncture in the management of musculoskeletal pain, including LBP, but none used acupuncture for back pain in pregnant women. Participating physiotherapists attended a 3-day training programme with the study team, which included education about pregnancy and pregnancy-related LBP, patient assessment, intervention protocols for the standard care and acupuncture interventions and trial procedures. Physiotherapists completed three questionnaires, before and immediately after the training programme and at the end of the EASE Back pilot trial. Self-confidence in assessing and managing pregnant women with back pain was assessed using four items (adapted from [16]) where responses are summed (after reverse-coding the first item) to give a self-confidence score (0–20); the higher the score the higher the confidence. Physiotherapists were also asked to indicate their clinical management of a typical patient presented as a written patient case vignette. In addition, following the end of all treatment in the pilot trial, physiotherapists were invited to a feedback workshop to gain their views on participation in the pilot trial in order to inform a future full RCT.

### Interventions

Participants were randomised to one of three treatment arms: standard care, standard care plus true acupuncture or standard care plus non-penetrating acupuncture.

Standard care (SC): the SC intervention was informed by the results of our studies undertaken prior to this pilot trial [8, 11, 12]. SC comprised a high quality and comprehensive self-management booklet, which was posted to participants following randomisation. Women could also access EASE Back physiotherapy care if needed, consisting of an individualised assessment and two to four treatment sessions over 6 weeks, with the episode of care ‘left open’ until the end of the pregnancy. Usual physiotherapy treatments could be used in face-to face physiotherapy sessions and might include advice, exercise approaches, heat, massage, manual therapy and issuing of pelvic supports/belts. Hydrotherapy, group treatments and acupuncture were not permitted within the protocol for SC alone. Women were not instructed to avoid other treatments, but their use of other treatments was monitored through self-report questions in the follow-up questionnaire.

Standard care plus true acupuncture (SC+TA): the same self-management booklet was posted to participants randomised to this arm. In addition, EASE Back trained physiotherapists delivered true acupuncture. This consisted of an individualised assessment followed by six to eight treatment sessions over 6 weeks with the acupuncture treatment lasting 20–30 min and manual stimulation of the needles aimed to elicit and maintain De Qi needling sensation. Depending on assessment findings, physiotherapists chose a minimum of 6 and a maximum of 10 bilateral points (between 12 and 20 points in total) using the principles of Western acupuncture and trigger point acupuncture from the list presented in Table 1.

**Table 1:** Acupuncture points and depth of needling in the true acupuncture arm

Point	Description	Depth of insertion/needle
Local points		
BL23	1.5 cun lateral to the spinous process of L2	30–40 mm/50 mm
BL24	1.5 cun lateral to the spinous process of L3	30–40 mm/50 mm
BL25	1.5 cun lateral to the spinous process of L4	30–40 mm/50 mm
BL26	1.5 cun lateral to the spinous process of L5	30–40 mm/50 mm
BL27	1.5 cun lateral to the spinous process of S1	30–40 mm/50 mm
BL28	1.5 cun lateral to the spinous process of S2	20–30 mm/40 mm
BL54	3 cun lateral to the spinous process of S4	50–70 mm/75 mm
BL31	Over the first sacral foramen	20–30 mm/40 mm
BL32	Over the second sacral foramen	20–30 mm/40 mm
BL33	Over the third sacral foramen	20–30 mm/40 mm
GB30	Over the piriformis muscle, at junction of lateral 1/3 and medial 2/3 of line joining sacral hiatus and greater trochanter of the femur	50–70 mm/75 mm
HJJ L4	One fingerbreadth lateral to the spinous process of L4	30–40 mm/50 mm
HJJL5	One fingerbreadth lateral to the spinous process of L5	30–40 mm/50 mm
All of the above points can be needled bilaterally. Tender points over the gluteus minimus and the pelvic rim can also be included.		
Distal points		
GB34	One fingerbreadth anterior and one inferior to the fibular head	25–30 mm/40 mm
ST36	3 cun below the joint line of the knee and a fingerbreadth lateral to the tibial crest	25–30 mm/40 mm
LR3	Just distal to the junction of the bases of the first and second metatarsal bones	25–30 mm/40 mm
LI4	On the highest point of the first dorsal interosseous muscle of the hand	20–30 mm/40 mm–30 mm
BL60	Midway between the lateral malleolus and the Achilles tendon	15–25 mm/30 mm
BL62	1 cun below the tip of the lateral malleolus	10–20 mm/30 mm
One or two of the above points are to be included in the treatment. Not necessary to needle bilaterally, unless the clinician judges it to be needed.		

The acupuncture protocol was informed by acupuncture texts, a national survey of acupuncture practice (Bishop et al. 2015) and previous studies (Elden et al. 2008 [18])

Standard care plus non-penetrating acupuncture (SC+NPA): to control for time and attention with physiotherapists and non-specific effects of acupuncture, a SC+NPA intervention arm was included. The non-penetrating needles look exactly like real needles, but the tip is blunted and the shaft of the needle slides in the handle, giving an illusion of penetration. The non-penetrating Streitberger needle has been used successfully in two previous trials of acupuncture [17, 18]. The same self-management booklet was posted to participants randomised to SC+NPA intervention arm. NPA was delivered by the same EASE Back trained physiotherapists and consisted of an individualised assessment followed by six to eight treatment sessions over a period of 6 weeks. The non-penetrating acupuncture was applied bilaterally over four bilateral points (eights acupuncture points in total) with treatments lasting 20–30 min.

For each participant, physiotherapists recorded full details of the treatment sessions including number, any non-attendance, the acupuncture points used, any sensations reported by participants during acupuncture treatments and any adverse events on case report forms. These case report forms also included a numerical rating scale to capture pain severity after each treatment.

### Data collection

Baseline data collection consisted of a baseline questionnaire and two objective self-administered tests of pain provocation to identify PGP [19]. Patient-reported outcomes were assessed at 8 weeks follow-up, to maximise the likelihood that most participants would have received their full course of treatment but would not have given birth. Non-responders to the 8-week follow-up mailing received a reminder mailing 2 weeks later. Attempts to collect a minimum outcome dataset by telephone occurred 2 weeks after the postal reminder.

Maternity records were reviewed following delivery to collect maternal and neonatal outcomes. Data on adverse events were collected on case report forms completed after each physiotherapy treatment from self-report of participants in the 8 weeks follow-up questionnaire and through maternity record reviews.

### Outcomes

All outcomes were included to inform a future full trial. No primary outcome was defined for this pilot trial, as the feasibility of use of a number of key pain and disability outcomes was investigated.

#### Process outcomes

These included recruitment rates, follow-up rates at 8 weeks, attendance at treatment sessions, treatment protocol adherence and completion rates on key outcome measures, as well as floor and ceiling effects of key outcomes measures.

#### Clinical outcomes

These included measures of pain, everyday function and general health at 8-week follow-up as well as the overall global rating of change since baseline, whether or not the woman was still pregnant (and if yes, number of weeks gestation), treatment credibility, satisfaction with the treatment package received, satisfaction with the results from treatment and side effects experienced.

### Economic outcomes

Patient-level data on costs were collected to conduct a preliminary economic evaluation and inform the design of a cost-utility analysis alongside a future full trial. NHS resource use data included GP consultations, number of treatment sessions attended by each participant, visits to other health care professionals, outpatient appointments, investigations or treatments and inpatient stays related to their back pain during pregnancy. To assess broader societal economic consequences, self-reported data on employment status, occupation, time off work and reduced productivity at work (presenteeism) were collected over the 8 weeks study period. Patient borne costs were over the counter medicines and devices and costs associated with attending trial treatment sessions, namely travel costs, time off work and child care costs. Quality of life was assessed through the EQ-5D-5L questionnaire [20].

### Maternal/neonatal outcomes

These were collected from maternity records for all participants and included gestational week at time of birth, live births, length of labour (and second stage of labour), if labour was induced, mode of birth, whether the woman had an episiotomy or a perineal tear, estimated blood loss at birth, antenatal and postnatal haemoglobin count, pain relief during labour, baby’s gender, birth weight, Apgar scores at 1 and 5 min and whether the baby was admitted to the neo-natal unit.

### Adverse events

Data on adverse events (AEs) were collected through case report forms completed after each physiotherapy treatment, through self-report of participants in the 8-week follow-up questionnaire and through maternity record reviews. Expected AEs from the acupuncture interventions include drowsiness/light headedness, nausea/vomiting, fainting, bruising at needle sites, feeling hot/burning, headaches and transient pain at needle sites. We defined serious adverse events (SAEs) of death, hospitalisation, significant disability or incapacity, any life-threatening circumstance (to the woman or developing child) or any other medically significant occurrences that were potentially attributable to the trial procedures or treatments. SAEs were identified via treating physiotherapists.

A summary of the outcomes is shown in Table 2.

**Table 2:** EASE Back pilot trial outcome measures

Domains	Description			
Process outcomes	Recruitment rates, follow-up rates at 8 weeks (overall and in each treatment arm), attendance at treatment sessions, treatment protocol adherence, key outcome measure completion rates, floor and ceiling effects of key outcomes measures			
Maternity record data				
Maternal outcomes	Gestation week at delivery, live births, length of labour (and second stage of labour), induction required, mode of delivery, episiotomy or a perineal tear, estimated blood loss at birth, antenatal and postnatal haemoglobin count, pain relief during labour			
Neonatal outcomes	Gender, weight, Apgar score at 1 and 5 min, admittance to neonatal unit			
Questionnaire data		Timepoint		
		Baseline	8 weeks full	8 weeks MDC^a^
Socio-demographics	Age, education (highest qualification), marital status, social support (living alone), number of children and pregnancies, job title, physical demands of work, overall work satisfaction, current body mass index (BMI) and pre-pregnancy BMI	✓	✗	✗
Work performance	Work status, time taken off work because of LBP, performance at work	✓	✓	✗
Pain location	Body chart (coded into LBP only, LBP with anterior PGP, LBP with pain in other bodily regions, or LBP with anterior PGP and pain in other bodily regions)	✓	✗	✗
Pain	Duration of current episode	✓	✗	✗
	Pain index (Dunn et al. 2010 [23]): mean of 3 numerical rating scales (least, usual and current pain)	✓	✓	✓
	Pain intensity before bed (Elden et al. 2008 [18])	✓	✓	✗
Sleep	Frequency of pain preventing sleep onset and waking at night	✓	✓	✗
Impact of pain on daily activities	Oswestry Disability Index (Fairbank et al. 1980 [24]) and the Pelvic Girdle Questionnaire (Stuge et al. 2011 [25], Grotle et al. 2012 [26])	✓	✓	✓
Quality of life	EuroQol EQ-5D-5L (Herdman et al. 2011 [20]) and SF-12 (Ware et al. 1996 [27])	✓	✓	✗
Use of medication, treatments or appliances	Over the counter and prescribed	✓	✓	✗
Treatment specific preferences and expectations	Preference for EASE Back treatments, expectation of effect of EASE Back treatments (11 point NRS no expected change–full recovery)	✓	✗	✗
Global improvement	Global rating of change since baseline—1 item 6 response options (complete recovery–much worse)	✗	✓	✓
Pregnancy	If still pregnant, if pregnant weeks gestation	✗	✓	✓
EASE Back treatment related costs borne by participant	Time off work for appointments (paid, unpaid, self-employed), how work was covered, time impact of treatment on other activities (family/domestic responsibilities, leisure activities, housework, studying), child care costs, accompanied to appointments, mode of transport and transport costs	✗	✓	✗
Health care utilisation	Consultations, investigations and treatments	✗	✓	✗
Treatment credibility	Confidence treatment helped pain, confidence in recommending treatment to others, perception of logic of treatment, perceived usefulness of treatment for alleviating other complaints	✗	✓	✗
Patient satisfaction	Satisfaction with EASE Back treatment package received, satisfaction with results of treatment	✗	✓	✗
Perceived side effects of treatment	Any side effects due to treatment and checklist of symptoms	✗	✓	✓
Objective tests				
Self-administered objective tests	Participant generated thigh thrust (P4) and bridging test with leg extension (Olsen et al. 2009 [19])	✓	✗	✗

MDC is minimum data collection where only key outcomes were collected from non-responders

### Blinding

Women randomised to standard care plus either true or non-penetrating acupuncture were blinded to which type of acupuncture they received. Physiotherapists providing the treatments could not be blinded but were not involved in any data collection procedure. Staff dealing with baseline and outcome data collection were blind to the intervention allocation. In addition, analyses of patient-reported outcomes at 8 weeks follow-up were conducted and verified by blinded statisticians.

### Statistical analysis

As a pilot trial, the analysis was mainly descriptive to inform the design of a full trial. A statistical analysis plan was developed with and agreed by the Trial Steering Committee prior to the start of analysis. All statistical analysis was performed using Stata version 13 [21].

Descriptive statistics were used to summarise key process and outcome variables. A CONSORT flowchart [13] shows the flow of participants into the trial, numbers allocated to each treatment arm, those providing follow-up data and included in the analysis. The success of each recruitment method was assessed by summarising the number and proportion of participants identified and recruited through each method. Description of participant baseline characteristics allowed an assessment of similarity between treatment arms. Descriptive summaries of the treatments delivered by treatment arm were described to assess treatment delivery and adherence to the protocol. For evaluation of the physiotherapist training programme, self-confidence in assessing and treating women with pregnancy-related back pain and changes in the reported management of a typical patient were examined. Descriptive statistics were used to summarise key clinical outcomes for those with data at both baseline and follow-up for each treatment arm; the degree of missing data was assessed in relation to all potential outcome measures. To inform the possible choice of primary outcome for a full trial, the performance of two measures of physical function (the ODI and the PGQ) and one measure of pain severity (a 0–10 numerical rating scale) was considered. This included the amount of missing data at the item and scale levels, any evidence of floor or ceiling effects, the precision of the outcome measures based on the standard error of measurement and their responsiveness to change. A detailed comparison of these measures will be reported in a separate paper.

Additional information on obstetric birth/neonatal outcomes for those women who reach the time of delivery within the timescale of the trial, overall and by treatment arm were described to assess any impact of treatment arm allocation on gestation, obstetric or neonatal outcomes. Satisfaction with treatment and assessment of treatment credibility was described by treatment arm. Any AEs and any SAEs considered to be related to the study procedures or interventions were described.

#### Health economic evaluation

In the absence of standard practice regarding the type of economic evaluation to conduct alongside feasibility and pilot studies, a cost–consequence analysis was chosen, with the aim of informing the design of a cost analysis within a full trial. The SC+NPA arm of the trial was not included in the economic analysis; therefore, key results relate only to the costs and consequences for the SC and SC+TA arms of the trial. Costs for each trial arm were calculated for each broad cost category (health care costs, patient-incurred costs, productivity costs) and in a disaggregated form within each of these cost categories. The base-case cost analysis adopted a National Health Service (NHS) and personal social services (PSS) perspective. A broader costing perspective was considered in a sensitivity analysis, taking into account NHS/PSS costs, patients’ personal expenditure and costs associated with work loss. Quality of life scores at baseline and 8 weeks and total QALYs over the 8-week period were calculated using the EQ-5D 5L. Analyses were mainly descriptive, and all costs and outcomes are summarised using means and standard deviations. As part of the sensitivity analysis, the results were replicated from a healthcare and wider societal perspective.

## Results

**Fig. 1:**
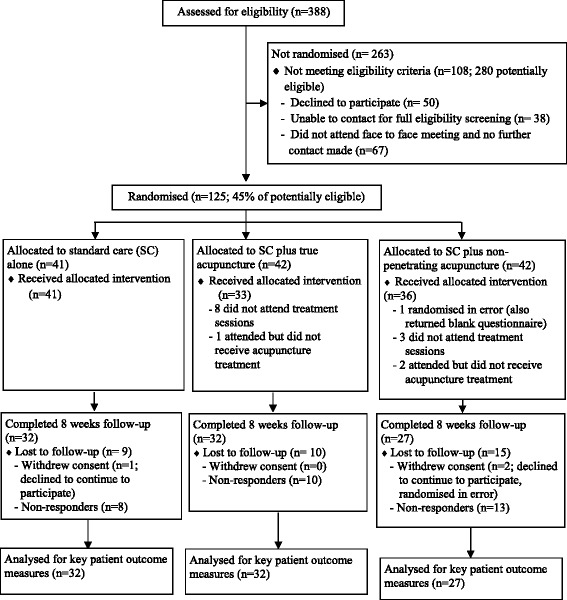
CONSORT diagram for the EASE Back pilot trial

Of 388 women assessed for eligibility over the 27-week recruitment period (April to October 2013), 108 did not meet the eligibility criteria. Of the 280 who were potentially eligible, 155 were either not assessed for full eligibility or were eligible but declined to participate and 125 women (45%) were recruited and randomised. One participant, in the SC+NPA arm, was randomised in error (they fulfilled the exclusion criteria for a high risk pregnancy) and was subsequently withdrawn prior to treatment. Overall, the 8-week follow-up rate was 73% (*n* = 91). Participant flow into the EASE Back pilot trial is shown in the CONSORT diagram in Fig. 1.

### Participants’ baseline characteristics

Participants’ baseline characteristics (Table 3) were largely similar across the three treatment arms. The mean age of the 124 participants who were randomised and completed a baseline questionnaire was 28 (SD 5.3) years and 53 (43%) were 24 or more weeks pregnant at the time of inclusion. More than half (68/124) had their pain for more than 6 weeks and the mean pain severity (Pain Index) was 4.6 (SD 1.7). About a third (36/124) had severe back pain-related disability and just over a quarter (38/144) had LBP and anterior PGP and pain in other parts of the body.

**Table 3:** Baseline characteristics of pilot trial participants

Baseline characteristics	Standard care	SC + true acupuncture	SC + non-penetrating acupuncture
	(*n* = 41)	(*n* = 42)	(*n* = 41)
Age (years), mean (SD)	27.8 (5.4)	28.1 (5.1)	29.0 (5.3)
Highest qualification: degree/postgraduate, *n* (%)	13 (31.7)	14 (33.3)	18 (43.9)
Gestation weeks at inclusion: 24+, *n* (%)	17 (41.5)	18 (42.9)	18 (42.9)
Married, *n* (%)	16 (39.0)	19 (45.2)	19 (46.3)
No. of children, *n* (%)			
0	16 (39.0)	20 (47.6)	14 (34.2)
1	17 (41.5)	13 (31.0)	17 (41.5)
2 or more	8 (19.5)	9 (21.4)	10 (24.4)
Working, *n* (%)	28 (68.3)	27 (64.3)	32 (78.1)
Physical demands of current/most recent paid job: heavy/very heavy, *n* (%)	12 (29.3)	13 (32.5)	11 (26.8)
Taken time off during the current pregnancy because of back pain^a^, *n* (%)	8 (28.6)	9 (33.3)	15 (46.9)
Back pain interference with performance at work (0–10 NRS) ^a, b^, mean (SD)	5.0 (2.5)	4.9 (2.6)	5.0 (3.1)
Work satisfaction (0–10 NRS)^a, c^, mean (SD)	6.6 (2.7)	5.4 (1.9)	6.6 (2.3)
Pain location (manikin), *n* (%)			
Low back pain only	7 (17.1)	9 (21.4)	7 (17.1)
Low back pain with anterior PGP	8 (19.5)	6 (14.3)	4 (9.8)
LBP with anterior PGP and pain elsewhere	10 (24.4)	14 (33.3)	12 (29.3)
LBP and pain elsewhere	16 (39.0)	13 (31.0)	18 (43.9)
Duration of episode: >6 weeks, *n* (%)	23 (56.1)	20 (47.2)	25 (61.0)
Pain index^d^, mean (SD)	4.5 (1.6)	4.5 (1.5)	4.6 (1.8)
Pain intensity before going to bed, mean (SD)	6.8 (1.9)	6.8 (1.8)	7.0 (2.2)
Woken up most/every night by pain, *n* (%)	16 (39.0)	15 (35.7)	17 (41.5)
Oswestry Disability Index (0–100)^e^, mean (SD)	34.8 (11.2)	32.9 (13.7)	35.7 (13.6)
Oswestry Disability Index (categorised), *n* (%)			
Minimal disability (0–20%)	5 (12.2)	8 (19.0)	4 (9.8)
Moderate disability (21–40%)	24 (58.5)	20 (47.6)	23 (56.1)
Severe disability (40–60%)	12 (29.3)	13 (31.0)	11 (26.8)
Crippled^f^ (61–80%)	0 (0.0)	1 (2.4)	3 (7.3)
Pelvic Girdle Questionnaire (0–100)^g^, mean (SD)			
Total	56.9 (16.0)	48.7 (17.2)	54.2 (17.3)
Activity subscale	54.6 (17.0)	46.7 (18.1)	52.7 (18.1)
Symptom subscale	65.4 (17.1)	56.1 (17.2)	59.3 (19.9)
Pre-pregnancy BMI, *n* (%)			
Normal/underweight	16 (40.0)	19 (47.5)	12 (31.6)
Overweight	8 (20.0)	11 (27.5)	17 (44.7)
Obese/morbidly obese	16 (40.0)	10 (25.0)	9 (23.7)
Taking prescribed medication, *n* (%)	7 (17.1)	5 (11.9)	5 (12.2)
Treatment preferences: Yes, *n* (%)	10 (24.4)	8 (19.1)	10 (24.4)
SF12—PCS, mean (SD)	36.4 (7.6)	38.2 (8.0)	37.0 (9.7)
SF12—MCS, mean (SD)	46.6 (10.1)	44.7 (12.1)	48.1 (11.7)
Self-assessed P4 test: familiar pain produced or increased in lumbosacro-iliac area^h^, *n* (%)	25 (75.8)	26 (81.3)	22 (66.7)
Bridging with extension of leg: familiar pain produced or increased in lumbosacro-iliac area^h^, *n* (%)	25 (89.3)	24 (80)	23 (82.1)
Positive on both P4 test and bridging test, *n* (%)	17 (41.5)	21 (50.0)	16 (39.0)

*SF12-PCS* Physical Component Scale, *SF12-MCS* SF12 Mental Component Scale (scales are based on a ‘normalised’ general population average of 50 with standard deviation of 10)

^a^Applies to only those in paid job

^b^Higher mean score implies more interference

^c^Higher mean scores imply more satisfaction

^d^Mean of three numerical rating scale for least, usual and current pain

^e^Oswestry Disability Index has ten sections with scores ranging from 0 to 5 in each section, item scores are summed and transformed to yield a score of 0 to 100 where 100 is the worst possible score

^f^‘Crippled’ is the term used in the original classification of the Oswestry Disability Questionnaire

^g^Pelvic Girdle Questionnaire—items are scored on a four-point scale, and item scores are summed and transformed to yield a score of 0 to 100 where 100 is the worst possible score

^h^Percentage applies to those who were able to perform the test

### Recruitment and follow-up

Recruitment lasted for a total of 27 weeks from April to October 2013. The number and proportion of women identified and recruited through the six methods are presented in Table 4. The brief screening questionnaire for back pain given to women as they attended their routine 20-week ultrasound scan appointment was the most successful method, followed by community midwives providing women with an EASE Back study card and women self-referring following the local awareness-raising efforts.

**Table 4:** Summary of the success of the six methods of identification and recruitment

	1. Research midwives in usual antenatal clinics	2. Screening questionnaire at 20-week scan	3. Screening of women’s health physiotherapy referrals	4. Research midwives or obstetricians give women EASE Back card	5. Community midwives or GPs give women EASE Back card	6. Self-referral	Total
Total number of women identified *n* (%)	28 (7.2)	199 (51.3)	49 (12.6)	3 (0.8)	73 (18.8)	36 (9.3)	388
Potentially eligible *n*	19	157	29	2	52	21	280
Randomised *n* (% of total women identified)	12 (42.9)	48 (24.1)	8 (16.3)	2 (66.7)	39 (53.4)	16 (44.4)	125 (32.2)
Randomised *n* (% of potentially eligible)	12 (63.2)	48 (30.6)	8 (27.6)	2 (100.0)	39 (75.0)	16 (76.2)	125 (44.6)

Column headings are the six methods of identifying and recruiting women

The 8-week follow-up rate was 73% (91/124). There was a slight imbalance in the follow-up rates between treatment arms with the SC plus non-penetrating acupuncture arm having the lowest response rate at 8 weeks (66%) compared to 80 and 76% in the SC and SC plus acupuncture arms, respectively. Only 14% of the women had given birth by the time they returned the 8-week follow-up questionnaire.

### Blinding

During telephone minimum data collection (MDC), three cases of unblinding of the research nurse conducting the calls were recorded out of the 70 MDC calls conducted. All three had been randomised to receive SC plus true acupuncture.

### Treatment delivery and adherence

Descriptive summaries of the treatments delivered are shown in Table 5. Only 4 of the 41 participants (10%) randomised to SC accessed face-to-face physiotherapy. Eight participants randomised to SC+TA, and three in the SC+NPA arm failed to attend for any treatment and therefore received no acupuncture. The number of treatments participants received was lower than anticipated, mainly due to the high levels of non-attendance for treatment appointments. Whilst the average number of treatment sessions in both arms receiving acupuncture was 6 (in line with the specified protocols), 13 (38%) in the SC+TA arm and 9 (23.7%) in the SC+NPA arm attended for fewer than 6 sessions.

**Table 5:** Summary of treatments delivered

	Treatment arm		
	Standard care (*n* = 41)	SC + true acupuncture (*n* = 42)	SC + non-penetrating acupuncture (*n* = 41)
Participants			
Number of participants who booked appointment to see a physiotherapist	4	37	41
Total number of participants who attended at least one treatment session with a physiotherapist	4	34	38
Total number of participants who received acupuncture treatment	0	33	36
Number of treatment sessions provided (per participant), median (IQR)	2 (1, 4)	6 (3, 7)	6 (6, 7)
Number of treatment sessions per participant (categorised), *n* (% of no. of participants seen)			
1–5	4 (100)	13 (38.2)	9 (23.7)
6–8	0 (0.0)	17 (50.0)	27 (71.1)
9–11	0 (0.0)	4 (11.8)	2 (5.3)
Number of treatments per participant in line with protocols^a^, *n* (% of number of participants who attended at least one treatment session)	2 (50.0)	17 (50.4)	27 (71.1)
Treatment sessions: total number planned	13	240	273
Total number of treatment sessions provided (% of total planned)	9 (69.2)	189 (78.8)	224 (82.1)
Total number of sessions that were not attended—DNAs/UTAs (% of total planned)	4 (30.8)	51 (21.3)	49 (17.9)
Total number of sessions attended where acupuncture was provided, *n* (% of attended)	0 (0.0)	163 (86.2)	197 (87.9)
Pain severity after treatment (0–10), mean (SD)	6.0 (0.8)	1.9 (2.0)	2.3 (2.3)
Number (%) of participants who received the treatment at least once during the course of treatment for participants who attended at least one treatment session with a physiotherapist	*n* = 4	*n* = 34	*n* = 38
Assessment/reassessment	4 (100.0)	34 (100.0)	38 (100.0)
Education and advice	4 (100.0)	34 (100.0)	38 (100.0)
Tubigrip provided with instruction	3 (75.0)	17 (50.0) (50.0)	19 (50.0)
Pelvic support belt provided with instruction	2 (50.0)	9 (26.5)	7 (18.4)
Heat therapy used in clinic	0 (0.0)	3 (8.8)	2 (5.3)
Massage therapy used in clinic	0 (0.0)	8 (23.5)	8 (21.05)
Manual therapy used in clinic	0 (0.0)	0 (0.0)	2 (5.3)
Supervised exercises in clinic	1 (25.0)	24 (70.6)	20 (52.6)
Home exercises given/reviewed	4 (100.0)	29 (85.3)	33 (86.8)
Issued walking aids	2 (50.0)	2 (5.9)	5 (13.2)
Number (%) of participants offered an exercise approach at least once during the course of treatment for participants who attended at least one treatment session with a physiotherapist	*n* = 4	*n* = 34	*n* = 38
Transversus abdominus muscle strengthening	2 (50.0)	24 (70.6)	25 (65.8)
Pelvic floor muscle strengthening	2 (50.0)	19 (55.9)	21 (55.3)
Pelvic tilt exercise	3 (75.0)	26 (76.5)	21 (55.3)
Gluteal muscle strengthening	2 (50.0)	25 (73.5)	22 (57.9)
Lower back/pelvic stretching	1 (25.0)	15 (44.1)	10 (26.3)
Advice/signposting to general physical activity opportunities	2 (50.0)	8 (23.5)	12 (31.6)
Other exercises^b^	1 (25.0)	13 (38.2)	15 (39.5)

*DNA* did not attend, *UTA* unable to attend, *SC* standard care, *TA* true acupuncture, *NPA* non-penetrating acupuncture

^a^2 to 4 sessions for SC, 6 to 8 for SC + acupuncture

^b^Other exercises included advice on aquanatal classes, increase repetitions, correcting standing posture, gym ball exercise, thoracic rotation, lumbar spine extension, obliques exercises and reviewing exercises in the EASE Back booklet

### Physiotherapy training programme and experience of delivering trial treatments

The training programme for participating physiotherapists increased their self-confidence in assessing and managing women with pregnancy-related LBP. Median self-confidence pre-training (measured on a 0–20 scale) was 8.0 (IQR 6.0, 11.0) and after training was 16.0 (IQR 16.0, 17.0) which was unchanged at the end of the trial.

For treatment of the typical patient presented in the vignette, increase in the reported use of written advice on self-management, advice to continue with everyday activities, advice about safe pharmacological treatment options and advice about the use of home massage were seen following the EASE Back training programme. Supervised exercise, strengthening exercise, pelvic floor exercise and acupuncture all increased following the training programme.

In the feedback workshop, physiotherapists made the following recommendations for a full trial:

- adding uncontrolled epilepsy, allergy to metal and allergy to latex (constituent of the elasticated bandage used as pelvic support) to the list of exclusion criteria for a future full RCT
- adding extra emphasis that participation in the trial meant attending for a full course of treatment sessions as arranging suitable appointments and non-attendance rates were problematic
- including more content on management of pubic symphysis pain (anterior PGP) in the physiotherapists’ training programme.

The participating physiotherapists reported that the following aspects worked well in the pilot and should be included in a future full RCT: the offer of evening treatment times to participants; scheduling participants’ whole treatment course into physiotherapists’ diaries; ensuring flexibility in staff providing EASE Back treatments to cover holidays; a minimum of two physiotherapists’ trained in EASE Back at each treatment site to provide peer support and sufficient capacity to treat participants; the mentoring support and supervision from the acupuncture trainer; and 1 hour new patient appointment slots to ensure there was sufficient time to provide advice, start the exercise programme and deliver acupuncture.

### Missing data on key clinical outcomes, evidence of floor or ceiling effect and responsiveness

There were no missing data for pain severity at both baseline and follow-up for those who returned the questionnaires, but there were minimal amounts of missing data for both ODI and PGQ. The proportion of the questionnaire items with missing data for all participants at baseline was 2.8 and 6.2% for the ODI and PGQ, respectively. At follow-up, the proportion was 7.8 and 8.7% for the ODI and PGQ, respectively. No floor or ceiling effects were shown in the total score of either the ODI or the PGQ. All the instruments showed good responsiveness, with all the area under a receiver operating characteristic curve more than 0.7. The detailed testing of these measures is reported separately.

### Exploratory analysis of key clinical outcomes

The descriptive statistics (mean and SD) of the key clinical outcomes (pain and disability outcomes) at baseline and 8 weeks follow-up are presented in Table 6. Table 6 also shows the differences in the mean scores from baseline. Overall, there was a reduction in pain and disability scores from baseline to 8 weeks follow-up. Greater reduction was observed in the SC+TA and SC+NPA arms compared to SC alone.

**Table 6:** Descriptive analysis of key clinical outcomes for those with data at both baseline and follow-up (*n* = 91)

Key outcomes	Baseline			8 weeks follow-up			Difference in mean score (or %) from baseline (baseline–8 weeks score) (95% CI)		
	Treatment arms			Treatment arms					
	SC	SC+TA	SC+NPA	SC	SC+TA	SC+NPA	SC (*n* = 32)	SC+TA (*n* = 32)	SC+NPA (*n* = 27)
ODI score (0–100), mean (SD)	34.1 (11.4)	29.1 (10.9)	33.9 (13.6)	32.2 (18.8)	21.3 (17.7)	26.2 (14.4)	1.9 (−5.1, 8.8)	7.8 (1.6, 14.0)	7.7 (2.9, 12.5)
PGQ (0–100), mean (SD)									
Total	57.3 (16.0)	45.9 (16.5)	51.9 (17.6)	52.2 (28.4)	31.9 (21.8)	40.3 (21.8)	5.1 (−5.1, 15.2)	14.0 (6.9, 21.2)	11.6 (5.2, 18.0)
Activity subscale	55.6 (16.9)	44.1 (17.6)	50.2 (18.9)	51.5 (29.4)	31.3 (22.1)	40.0 (23.4)	4.1 (−6.4, 14.6)	12.8 (5.4, 20.3)	10.1 (3.5, 16.8)
Symptom subscale	63.0 (16.8)	52.6 (16.4)	58.0 (19.6)	54.4 (26.5)	33.8 (22.4)	40.2 (20.2)	8.6 (−1.9, 19.0)	18.8 (11.1, 26.5)	17.8 (9.7, 25.8)
Pain index^a^	4.4 (1.6)	4.3 (1.5)	4.2 (1.9)	4.2 (2.2)	2.4 (2.2)	2.2 (1.5)	0.2 (−0.8, 1.2)	1.8 (1.1, 2.5)	2.1 (1.4, 2.9)
Pain intensity before going to bed: 0–10 NRS, mean (SD)	6.5 (2.1)	6.5 (2.1)	6.7 (2.2)	6.5 (2.8)	3.7 (2.8)	3.5 (2.3)	0.04 (−1.5, 1.6)	2.7 (1.4, 4.0)	3.2 (1.8, 4.5)
Woken up most/every night by LBP, *n* (%)^c^	9 (37.5)	6 (22.2)	7 (31.8)	9 (37.5)	4 (14.8)	1 (4.6)	0.0 (−27.4, 27.4)	7.4 (−13.2, 28.0)	27.3 (6.0, 48.6)
SF12-PCS, mean (SD)	36.2 (8.5)	38.7 (7.5)	37.6 (9.6)	36.5 (10.6)	41.4 (11.5)	43.3 (9.5)	−0.3 (−5.5, 4.9)	−2.8 (−6.2, 0.6)	−5.7 (−10.7, −0.7)
SF12-MCS^b^, mean (SD)	49.1 (8.8)	47.7 (12.7)	51.4 (10.3)	49.1 (8.1)	48.4 (13.8)	50.2 (12.3)	0.03 (−4.1, 4.1)	−0.2 (−4.2, 3.9)	1.2 (−5.3, 7.7)

*ODI* Oswestry Disability Index, *PGQ* Pelvic Girdle Questionnaire, *SF12-PCS* Physical Component Scale, *SF12-MCS* Mental Component Scale (scales are based on a ‘normalised’ general population average of 50 with standard deviation of 10)

^a^Mean of three numerical rating scale for least, usual and current pain

^b^Higher scores indicate better quality of life so negative sign in the difference indicate improvement in quality of life

^c^Differences are percentages of those who reported waking up at night at baseline and at follow-up

The mean scores (with 95% CI) at 8 weeks follow-up for each treatment arm adjusted for baseline scores and other baseline covariates are shown in Table 7. After adjusting for the baseline covariates, the mean outcome scores at 8 weeks follow-up were higher (worse outcome) in the SC arm compared to the true and non-penetrating acupuncture arms suggesting the intervention effects are feasible and worthwhile to pursue in a main trial.

**Table 7:** Adjusted estimates (estimated marginal means and 95% CI) of key outcomes at 8 weeks follow-up for each treatment arm separately

Key outcomes	Outcome mean (95% CI) adjusted for baseline scores only			Outcome mean (95% CI) adjusted for baseline scores and other covariates^d^		
	Treatment arms			Treatment arms		
	SC	SC+TA	SC+NPA	SC	SC+TA	SC+NPA
ODI score^a^ (0–100)	31.2 (25.5, 36.9)	23.2 (17.5, 28.8)	25.2 (19.1, 31.3)	31.3 (25.5, 37.0)	23.3 (17.5, 29.0)	25.0 (18.8, 31.3)
PGQ^b^ (0–100)						
Total	48.3 (40.5, 56.2)	35.8 (28.0, 43.5)	40.1 (31.8, 48.3)	48.3 (40.5, 56.1)	36.7 (29.0, 44.4)	40.0 (30.7, 47.2)
Activity subscale	47.6 (39.5, 55.7)	35.3 (27.3, 43.2)	39.9 (31.4, 48.4)	47.7 (39.7, 55.7)	36.2 (28.3, 44.2)	38.6 (30.1, 47.1)
Symptom subscale	52.0 (44.1, 60.0)	36.2 (28.3, 44.0)	40.1 (31.7, 48.5)	52.4 (44.4, 60.3)	36.0 (28.1, 43.8)	40.0 (31.5, 48.4)
Pain severity (pain index)^c^	4.1 (3.4, 4.8)	2.5 (1.8, 3.2)	2.1 (1.4, 2.9)	4.1 (3.4, 4.7)	2.6 (1.9, 3.2)	2.1 (1.3, 2.8)

^a^Oswestry Disability Index scores range from 0 to 100 with higher scores indicating greater disability

^b^Pelvic Girdle Questionnaire scores range from 0 to 100 where 100 is the worst possible

^c^Mean of three numerical rating scale for least, usual and current pain score

^d^Adjusted for baseline outcome score, age, gestation week and baseline pain severity (for the disability outcomes) and ODI score for the pain severity outcome

### Obstetric birth/neonatal outcomes

All women who participated in the pilot trial had live births (mean gestation 40 weeks (SD 1.5)). Labour duration was similar across treatment arms, as was the need to be induced, the mode of delivery, the proportion of women who either had an episiotomy or a perineal tear, and the mean estimated blood loss at birth. Pain relief used during labour was the same across all three treatment arms. Neonatal outcomes were also very similar across all three treatment arms. In total, only three babies (2.5%) were admitted to the neo-natal unit and 95% or more of all babies had an Apgar score of 7–10 5 min after birth.

### Treatment credibility and satisfaction with care

Overall, about 60% (43/71) of participants who completed full follow-up questionnaire were either very or quite confident that the information or treatment they received helped their LBP problem, 3/24 (13%), 22/27 (82%), and 18/22 (86%) in the SC, SC+TA and SC+NPA arms, respectively. In total, 69% (49/71) reported they would be very or quite confident to recommend the information/treatment to a friend who suffered from a similar problem; however, the proportions rating treatment credibility as high were much lower in the SC alone arm (7/24 = 29%). Levels of satisfaction with the treatment package received and the results from treatment were high overall (65%) but lower in the SC arm (17%) compared to SC+TA (85%) and SC+NPA (96%).

### Health economic evaluation

Complete baseline and 8 weeks economic data were available for 73 participants (24 in SC, 27 in SC+TA and 22 in SC+NPA); this represented 59% of the sample. The analysis focused on the 51 participants receiving SC versus SC+TA, which informed the base-case analysis. Table 8 shows the disaggregated mean (SD) costs for each treatment arm. The cost of treatment was reported as part of the total cost of each trial arm. The principle aim of the cost-consequence analysis was to look at all the costs of the two interventions (SC alone versus SC+TA) and compare these with the corresponding outcomes in terms of QALYs gain.

**Table 8:** Mean per participant costs by treatment arm over 8 weeks

Complete-case analysis	SC	SC+TA
	*n* = 24 Mean £ (SD)	*n* = 27 Mean £ (SD)
Intervention in the trial	11.52 (43.2)	149.43 (71.14)
GP visit(s)	12.75 (29.8)	17.62 (39.3)
NHS midwife	6.25 (30.6)	16.66 (48.03)
Private midwife	0.00 (−)	3.55 (18.4)
NHS acupuncture	0.00 (−)	9.96 (51.76)
NHS physiotherapist	2.04 (10.0)	16.85 (69.2)
‘Other’ professionals in NHS	5.83 (28.6)	3.74 (19.43)
Prescriptions	1.76 (8.6)	0.54 (2.8)
‘Over the counter’ treatments	8.67 (14.3)	7.38 (21.7)
LBP related work absence^a^	98.36 (330.3)	258.86 (517.8)
LBP related reduced productivity	806.55 (769.58)	783.45 (762.7)
NHS/PSS cost	40.15 (90.78)	214.82 (191.92)
Incorporation of back pain-related healthcare resource use (*n* = 51)		
Healthcare cost, £^b^	48.82 (94.18)	225.77 (205.65)
Incorporation of back pain-related wider societal resource use (*n* = 51)		
Societal cost, £^c^	953.74 (921.14)	1268.08 (1207.24)

Values are mean (sd) number of consultations/investigations unless stated otherwise.

*SC* standard care, *SC+TA* standard care plus true acupuncture

^a^Includes both work absence due to LBP and work absence to attend treatment sessions

^b^Includes costs to the NHS, private health care costs and over-the-counter medication costs

^c^Includes healthcare costs and productivity related costs

Table 9 shows the EQ-5D scores at baseline and 8 weeks and the total QALYs in the two arms. Health-related quality of life increased at 8 weeks follow-up. At 8 weeks, we observed a lower EQ-5D score in participants in the SC arm and a higher score for the SC plus true acupuncture arm.

**Table 9:** EQ-5D scores by treatment arm (mean and SD)

Complete-case analysis	SC	SC+TA
Baseline	*n* = 41	*n* = 42
	0.649 (0.12)	0.651 (0.16)
8 weeks	*n* = 24	*n* = 27
	0.570 (0.23)	0.698 (0.22)
Total QALYs	0.096 (0.02)	0.106 (0.02)
Adjusted^a^	0.096	0.106

*SC* standard care, *SC+TA* standard care + true acupuncture

^a^Incremental QALY estimates following multiple regression-based adjustment for baseline scores on the EQ-5D

The results show that although the SC+TA intervention has the highest total NHS cost and health care costs compared to SC alone, it is the intervention that also achieves higher QALY gains. From an NHS/PSS perspective, the mean costs per woman were £215 for SC+TA and £40 for SC alone. These costs reflect the higher resource use attributable to the additional acupuncture sessions and the relatively small reported increase in utilisation of acupuncture, midwifery and physiotherapy in a few participants in that treatment arm. Therefore, acupuncture provided in addition to SC seems more effective in treating pregnant women with LBP, but this increased effectiveness comes at an increased cost.

### Adverse events

No serious adverse events (SAEs) attributable to the trial interventions or trial processes were reported. There were four potential SAEs reported by treating physiotherapists to the trial team (one in SC, two in SC+TA and one in SC+NPA); all were brief periods of hospitalisation or admission to the maternity assessment unit, for reasons other than those related to the trial or treatments. All women were discharged after brief monitoring and all resumed treatment.

### Minor adverse reactions

Minor adverse reactions were reported by physiotherapists on case report forms. All were expected minor adverse events, with the most common slight bleeding at the needle sites in 35 (21%) treatment sessions in the SC plus true acupuncture arms and in 1 (0.5%) treatment session in the SC plus non-penetrating acupuncture arm. Other expected minor adverse events which included pain on needle insertion, slight bruising, drowsiness and slight soreness which occurred in less than 4% of treatment sessions.

## Discussion

This pilot trial has shown that a future full RCT is feasible. The eligibility criteria, patient information, processes for consent and randomisation, treatment protocols and case report forms worked well in the pilot RCT and should be included in a future full trial.

### Key findings and design implications for a full trial

#### Identification and recruitment of women

We identified women using six methods, three of which were the most successful and should be used in a full trial. These were the brief screening questionnaire given to women as they attended their routine 20-week ultrasound scan appointment, community midwives providing women with an EASE Back study card and women self-referring to the trial team following local awareness-raising efforts. We recruited a greater than anticipated proportion (45%) of eligible women, bearing in mind the young age group, many of whom were working and had other children to care for. In addition, the upper limit of 31 weeks gestation resulted in only 14% of participants giving birth prior to 8 weeks follow-up meaning improvements could be attributed to the interventions rather than being driven by improvements once women have given birth.

#### Training of physiotherapists

We trained 14 physiotherapists to deliver the EASE Back interventions and provided mentoring support. Training of physiotherapists will be important in a full trial as although all the participating physiotherapists had completed national standards of training in acupuncture, none were using it to manage women with pregnancy-related LBP prior to the pilot RCT training. This reflected our previous findings during the development of this pilot RCT that highlighted physiotherapists lack of experience of using acupuncture with pregnant women and concerns about safety [11]. The questionnaires completed by the physiotherapists showed marked increases in their self-confidence in assessing and managing pregnancy-related LBP and increased use of recommended management approaches such as providing written information, advice on safe pharmacological options and strengthening and pelvic floor exercises with more supervision of exercise by the physiotherapist.

#### Interventions

Treatments were delivered broadly in line with specified protocols, although some women did not attend for their allocated treatments despite efforts to contact them and re-engage them in treatments. A choice of appointments for treatment was provided, including different treatment centres and day and evening appointments, and this level of flexibility will be essential in a full trial. In the acupuncture arms, additional treatment consisted mostly of education, advice and exercises, particularly strengthening and pelvic floor exercises. Small numbers of participants received pelvic supports, heat and massage. These additional treatments were in line with the protocol. Very few women in the SC only arm accessed face-to-face physiotherapy (4 of 41) even though this was an option in the protocol. The reasons for this are unclear and this could be further investigated in a full trial using qualitative methods.

#### Follow-up

The follow-up rate in the pilot trial was 73% at 8 weeks, slightly less than we had hoped. However, we did anticipate this, as we were asked to remove one follow-up reminder from our usual trial follow-up processes by the research ethics committee and whilst we appealed this, the request was upheld. If we used our usual trial follow-up procedure which would include a further reminder mailing and telephone follow-up of non-responders, a minimum of 80% follow-up is likely to be achieved. However, a longer reminder and follow-up period would likely result in a greater proportion of women having given birth by the time they provided follow-up data.

#### Choice of primary outcome for a main trial

The pilot trial included two measures of physical function (the ODI and the PGQ) and one measure of pain severity, to inform the choice of primary outcome for a future main trial. Several factors were taken into consideration in examining the performance of these measures. These included the amount of missing data at the item and scale levels; any evidence of floor or ceiling effects, the precision of the outcome measures based on the standard error of measurement and their responsiveness to change. The detailed testing of these measures is reported separately, but taking all aspects of the performance of the measures into account, the PGQ is the recommended primary outcome for a future main RCT.

#### Sample size for a full trial

A future full trial is likely to be best powered to detect moderate differences between SC versus SC plus true acupuncture (testing effectiveness). If a future full trial also includes a comparison between SC plus true acupuncture versus SC plus non-penetrating acupuncture (testing the added efficacy of needle penetration and manipulation) or a treatment course of exercise-based physiotherapy (providing an attention control and testing the added efficacy of acupuncture), we anticipate needing to power the trial to detect small differences between these interventions. Using the PGQ total score at 8 weeks follow-up as the primary outcome of a future main trial, analysis of covariance (ANCOVA) with baseline covariate adjustment (baseline PGQ total score, gestation week, age) as the main analysis, with the covariates having a combined *R*-squared of 0.5 with the response, 80% power and 5% two-tailed significance level, we estimate that approximately 200 participants per trial arm would be needed to detect a four-point difference between true and non-penetrating acupuncture arms, using a baseline SD of 18 and allowing for 20% loss to follow-up. Thus, a future two-arm trial would require approximately 400 participants and three-arm trial would require 600 participants. The standard deviation for the sample size calculation was derived from the pilot data but allowing for the uncertainty surrounding the pilot estimates (i.e. using an 80% upper one-sided confidence limit) [22].

We also tested ways to collect health economic data and conducted an exploratory health economic analysis. As the cost-consequence results reported here are based on a small sample size, a full trial would be required to conduct a fully incremental analysis to make any recommendations based on cost-effectiveness. One limitation with the exploratory analysis is that although care was taken to avoid double counting, some of the patients could have reported visits as part of the trial within the additional health care visits.

### Strengths and limitations

This is the only randomised trial of acupuncture and standard care for women with pregnancy-related back pain in the UK. The strengths of this pilot trial were that all the procedures to inform a full trial design were tested, including identification of eligible participants, recruitment, randomization, intervention delivery and short-term follow-up. Only a short-term follow-up was included in the pilot trial and so cannot inform the likely response to a longer term outcome, which would be desirable in a full trial. As discussed earlier, the 8-week follow-up rate was less than 80%, but the number of follow-up reminders we had originally planned to use was reduced by the research ethics committee. Although we are confident that an additional follow-up reminder would achieve a higher follow-up rate, attention needs to be given to follow-up procedures in a full trial. As stated throughout the paper, this was a pilot trial and was not powered for hypothesis testing of clinical and cost outcomes. The clinical outcomes and cost results should therefore be viewed as exploratory. Clinical and cost-effectiveness can only be assessed in a full trial.

## Conclusions

In conclusion, a future main RCT testing the additional benefit of acupuncture to standard care is feasible. A full RCT, with longer term follow-up, would provide evidence about the effectiveness of acupuncture and inform treatment choices for women with pregnancy-related LBP.

## Abbreviations

ANCOVA: Analysis of covariance
BMI: Body mass index
CI: Confidence interval
CTU: Clinical trials unit
DNA: Did not attend
GP: General practitioner
HTA: Health technology assessment
IQT: Interquartile range
LBP: Low back pain
MCID: Minimum clinically important difference
MCS: Mental component scale
MDC: Minimum data collection
NHS: National Health Service
NIHR: National Institute for Health Research
NRES: National Research Ethics Service
ODI: Oswestry Disability Index
PCS: Physical component scale
PGP: Pelvic girdle pain
PGQ: Pelvic Girdle Questionnaire
PSS: Personal social services
QALY: Quality adjusted life year
RCT: Randomised controlled trial
RS: Responsiveness statistic
SAE: Serious adverse event
SC: Standard care
SC+NPA: Standard care plus non-penetrating acupuncture
SC+TA: Standard care plus true acupuncture
SD: Standard deviation
SES: Standardised effect size
SF12: Short Form 12 item
SRM: Standardised response mean
TENS: Transcutaneous electrical nerve stimulation
UK: United Kingdom
UTA: Unable to attend
